# Supplementary material for: Genetic and physical interactions between the organellar mechanosensitive ion channel homologs MSL1, MSL2, and MSL3 reveal a role for inter‐organellar communication in plant development
Source: Plant Direct. 2019 Mar 4;3(3):e00124. doi: 10.1002/pld3.124 (PMC6508831; doi:10.1002/pld3.124)
Supplement: Supplementary file 1 [file PLD3-3-e00124-s001.pdf]

A

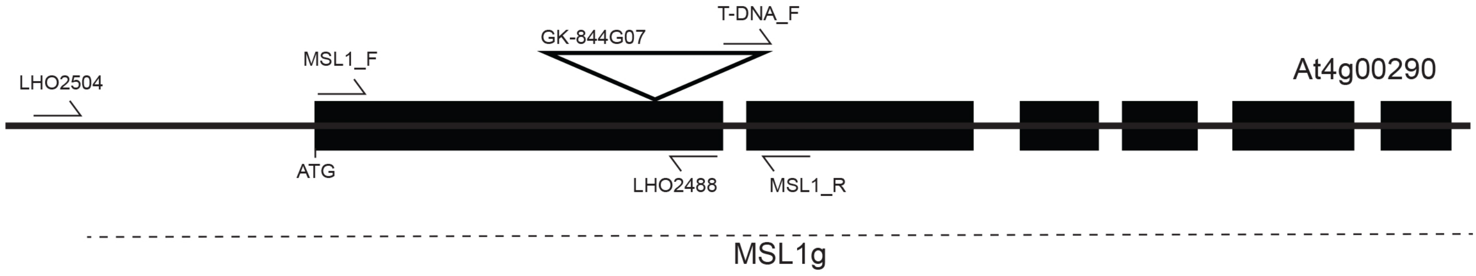

B

Target	MSL1_F, MSL1_R	T-DNA_F, MSL1_R	LHO2504, LHO2488
wild type *MSL1* genomic copy	+	-	+
MSL1g transgene	+	-	-
msl1-1 T-DNA insertion allele	-	+	-

C

Primer name	Sequence
MSL1_F	ATGGCCGGAGTTAGGTTATCGC
MSL1_R	CTTCTCCCTATCAAGCCCACC
T-DNA_F	CCCATTTGGACGTGAATGTAGACAC
LHO2504	CAAGAAGACGACGATGGCTGTGAAGGAACATGG
LHO2488	TAGGCATCACAACCCAAGCA

Figure S1. Primers involved in genotyping the genomic locus of *MSL1* in the presence of the *MSL1g* transgene. (A) Schematic of the *MSL1* gene and the location of primers used. Thick lines indicate exons and thin lines indicate introns. The inverted triangle indicates the insertion point of the T-DNA in the *msl1-1* mutant. Thin arrows (not to scale) indicate the approximate recognition sites of oligos used for genotyping. Dashed line indicates the sequence included in the *MSL1g* transgene. (B) Primer pairs used to distinguish the wild type genomic version of *MSL1*, the *MSL1g* transgene, and the *msl1-1* T-DNA insertion allele. (C) The sequences of primers used in (B) are shown.
