# Supplementary figures and images for: Genetic and physical interactions between the organellar mechanosensitive ion channel homologs MSL1, MSL2, and MSL3 reveal a role for inter‐organellar communication in plant development
Source: Plant Direct. 2019 Mar 4;3(3):e00124. doi: 10.1002/pld3.124 (PMC6508831; doi:10.1002/pld3.124)

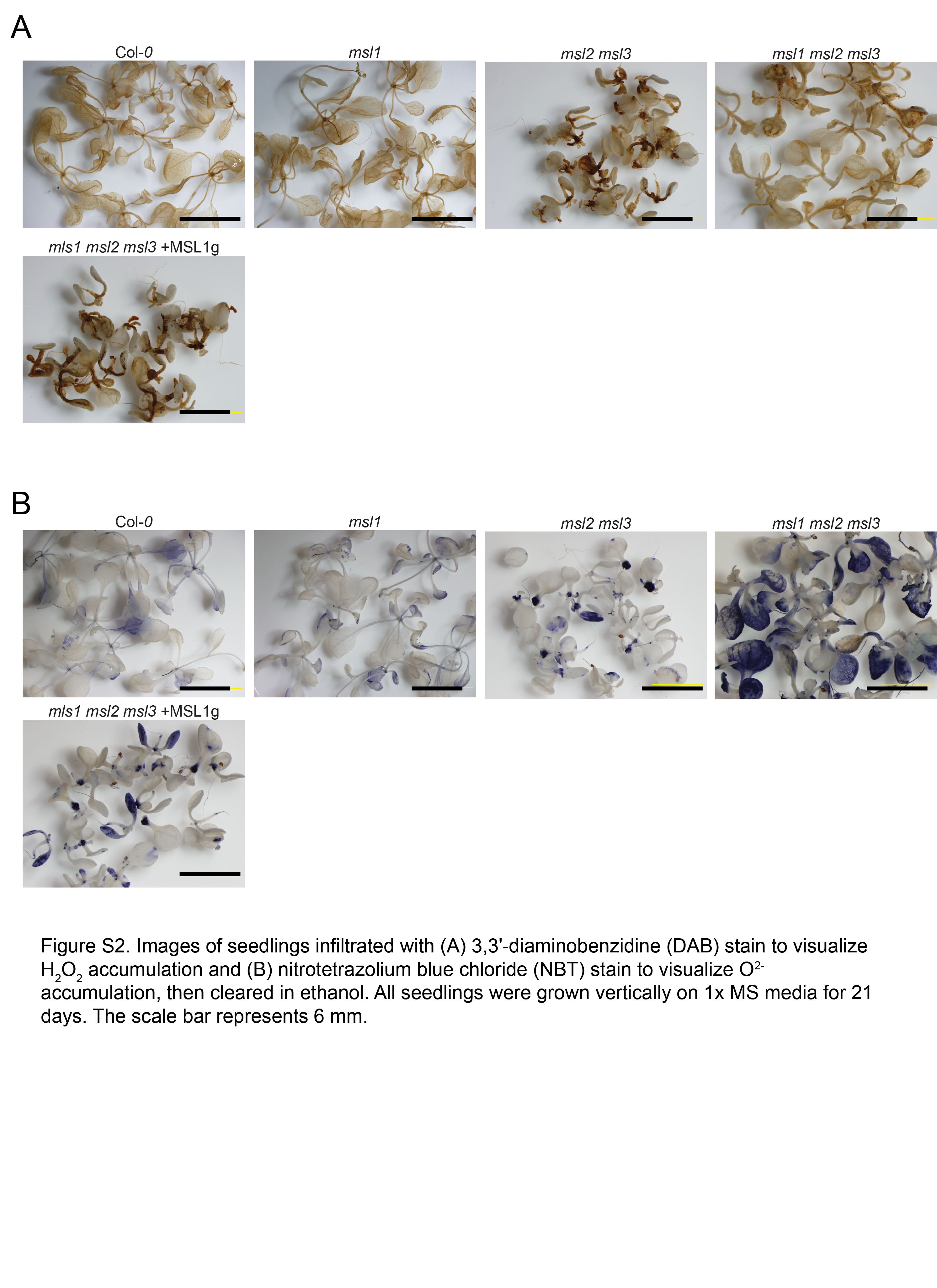

Supplement: Supplementary file 2 [file PLD3-3-e00124-s002.png]
