# Supplementary material for: Genetic and physical interactions between the organellar mechanosensitive ion channel homologs MSL1, MSL2, and MSL3 reveal a role for inter‐organellar communication in plant development
Source: Plant Direct. 2019 Mar 4;3(3):e00124. doi: 10.1002/pld3.124 (PMC6508831; doi:10.1002/pld3.124)
Supplement: Supplementary file 3 [file PLD3-3-e00124-s003.pdf]

RESPONSE TO REVIEWERS

Reviewer #1:

The manuscript present a very clear set of experiments in a concise manner. Other than a few typos, that can be easily fixed by a careful reading, the ms is quite straightforward to read. The conclusions are justified. The discussion is pertinent and while it is possible to ask for more speculative reasoning, I think the authors have done a commendable job in not over-interpreting their findings.
→*Thank you!*

Reviewer #2:

The conclusions ARE supported by the data, with the exception at present of the root growth differences.
The manuscript by Haswell and coworkers presents an interesting, intriguing set of observations which link proteins with previously demonstrated mechanosensitive, osmotic shock-protective roles in the inner membranes of chloroplasts with homologous proteins in the inner membrane of mitochondria.
The work focuses on the phenotypes of the respective mutants, or their combination, and is primarily descriptive. This is not a criticism, but it is the case that the mechanistic basis for the interactions between the mutations remains obscure. What the data do successfully prove, when taken together with evidence of subcellular localisation, is what is not their basis: the interactions are not due to a physical association between these proteins. With this in mind, as a reviewer it is difficult to avoid considering "what else" could have been done which could have shed light on the causes of the paradoxical phenotypes. Regrettably it is difficult to come up with such ideas, but there is one suggestion which can be offered: the central paradox is between the enhanced leaf phenotypes, in developed organs, and the suppressed callus formation or reactive oxygen species accumulation in shoot apical tissues. With a physical interaction ruled out, the next most likely explanation for an interaction between chloroplasts and mitochondria is a metabolic one. In metabolic terms, leaves are source tissues containing active chloroplasts exporting photosynthate. The shoot apex, on the other hand, possesses tissue with very high levels of expression of photosynthesis-associated genes,, but metabolically is still developing chloroplasts and acting as sink tissue. This drastic difference between chloroplast activities in older and younger tissues will surely lead to a fundamental difference in the relationship between chloroplasts and mitochondria in both cases. It is noteworthy that while the callus differentiation at the apex (previously shown to depend on chloroplast osmotic stress and its resulting oxidative stress) is most pronouncedd in the msl2 msl3 double mutant, and is ameliorated in the triple, the leaf developmental defects (morphologically suggestive of cell death) are worse in the triple and lesser in the double, and exactly the same contrast is observed for the accumulation of superoxide as reported by NBT staining: maximum in the double at the apex (Fig. 4) and in the triple in the developed leaves and cotyledons (Suppl. Fig. 2). The authors, possibly in future work, could consider exploring this difference. One easy-to-test metabolic interaction could be that initiated by photorespiration, which requires both chloroplast and mitochondrial participation. Improvement of leaf growth of triple mutants under high CO_2, and reduction of leaf lesions, would point to such a cause. Whether or not this could be carried out at present, this scenario seems worth discussing.

→We added the following to the discussion: "The leaf morphology and superoxide accumulation phenotypes of the msl2 msl3 double mutant were exacerbated in the msl1 msl2 msl3 background, while the callus production and root phenotypes were suppressed. One explanation for these differences is variation in the metabolic coupling of plastids and mitochondria. Eliminating MSL1, and thereby disrupting mitochondrial redox homeostasis, may have different effects in source tissues that are actively photosynthesizing and photorespiring (such as leaves) and sink tissues (such as roots and meristems) that are not."

The second issue worth addressing, the one current weakness in the data, refers to the root phenotypes in the double and triple mutants. While systemic signalling triggered by reactive oxygen species could explain the growth differences, the images provided in Fig. 5 show smaller young seedlings for the msl2 msl3 double mutant (or triple complemented with MSL1). Changes in the size of the source tissue would result in different amounts of available photosynthate, and as a result in different amounts of root meristematic activity. Thus such differences could be indirect actions of the mutations. Evidence for a direct action would come only from differences in root growth even in the presence of an exogenous sugar supply (reading of the methods indicates no sucrose supply in the medium).

→This is an useful suggestion. We added to the discussion "How directly a signal involving MSL1 leads to each of these phenotypes remains unclear. In the case of the root phenotypes, we note that msl1 msl2 msl3 seedlings were larger than msl2 msl3 seedlings (Figure 5). It is possible that the higher root length and number of lateral roots in msl1 msl2 msl3 may be an indirect effect of larger seedling size." We do not add sucrose to the media because growth in the presence of osmotic support (including sucrose) suppresses all other msl2 msl3 phenotypes.

A third, simpler issue: a better description of the "shooty" outgrowths in the triple mutants would help. From the images provided it is clear there is no callus formation, but what they actually look like is difficult to see. Are they like small seedlings, small internodes with terminal leaves? Are those leaves terminal? Do they arrest growth? A full description woudl require localisation of meristem identity genes, etc, as the authors have previously done, but such an effort seems unnecessary here, helping the reader with a better description of this reversal of "dedifferentiation" would be enough.

→We added the following to the text: "These outgrowths all arose from the region of the apical meristem and formed a terminal shoot. Outgrowths sometimes comprised a single leaf; other times clustered or branched leaves were observed." All of these phenotypes are shown in images in Figure 3D.

Small/textual issues:
Line 46: Neuhaus reference missing.
→fixed

Line 353: ...which in turn leads...
→fixed

Supp. Fig. 1: The logic of the transgene genotyping is unclear. Is the sequence complementary to the LHO2504 primer present in the transgene but not in genomic DNA? What is this primer's

target?

→This primer anneals to the genomic DNA but not to the transgene, giving a product from genomic DNA but no product from the MSL1 transgene nor from the msl1-1 allele when paired with the indicated reverse primer (shown in the table in Figure S1B). To clarify, we added a line that indicates the sequence included in the MSL1g transgene to Figure S1A and explained this in the figure legend.

Reviewer #3:

Genetic and physical interactions between the organellar mechanosensitive ion channel homologs MSL1, MSL2, and MSL3 reveal a role for inter-organellar communication in plant development
Josephine S. Lee et al. 2018

General comments:
Understanding plants inter-organellar communication is highly important in plant science, especially during plant development how different organelles such as mitochondria and plastids talk each other is very important for environmental stress to understand plant stress responses. The author very nicely frames necessary experiment and the manuscript is overall well written, and figures are generally well illustrated. However, minor error correction will make the manuscript more charming and insightful for the reader. Especially, I am recommending the Author should extremely care during literature citation, as most of the references are not homogenously formatted (example citation1 and 2, the Author write Journal name "Journal of Cell Science" "Current Opinion in Plant Biology" in full format, whereas, citation 3 and 4 the journal name is abbreviated - "Plant Physiol." "Front. Plant Sci."
Sometimes the author uses all first letter "cap" in journal title, on the other hand, it is a "small" letter. The author should make all the citation in homogenous and citation manager such as EndNote could helpful for this citation process.
→fixed

Scientific comments:
Signaling relationship between the two organelles could potentially change a series of developmental process, and the Autor already showed some changes in shoot apex and lateral roots. Does the author have any other cellular evidence (could be a confocal image) how that conformational change looks like during this process? It will be great if the Autor could show an elicitor (stress) that leads to change these plastids phenotypes, and why MSL1 seriously involved in that process?

→If we understand correctly, the reviewer is proposing the interesting experiment of looking at plastid and mitochondrial morphology in these different genetic backgrounds. These experiments are definitely a part of our future directions. However, to detect, quantify and validate any differences will be a substantial undertaking, and it is not possible to include it in the current manuscript.

Minor comments:
L-153: ~150 mmol m-2 s-1 (double check the unit)
→fixed

L-156: 16-h-light (remove the "-")
→fixed

L-157: 150 to 195 µmol m-1 s-1 (double check the unit)
→fixed

L-159: 21-day-old (remove the "-")
→fixed

L-161: 10 mM K2HPO4-KH2PO4 (double check "-")
Replaced with "10 mM potassium phosphate"

L- 166: mg/ml (should be mg ml_1)
→fixed

L-181 Arabidopsis Biological Resource Center (web portal reference missing)
→, https://abrc.osu.edu added

L-184: tryptophan, and uracil (remove "and" between)
→Changed to "Synthetic Minimal media lacking adenine, histidine, leucine, tryptophan, and uracil. This media was also supplemented with 150 µM Methionine"

L-189: (Bass:2002hg; Miller et al., 2003)} (delete "}" sign)
→fixed

L-193: (Basu and Haswell, 2017), indicated in yellow in Figure 1). (remove the reference from the bracket and rewrite straight with reference insertion)
→OK

L 205 to L-209: (as defined in (Haswell, 2007)); (as defined in (Lee et al., 2016)); (as defined in (Haswell and Meyerowitz, 2006)) - rewrite with straight sentence and keep those references in description section rather than figure legend, which will make reader confusion.

→We appreciate the reviewer's suggestion but prefer to include this important information in the figure legend. Different authors define the MscS domain differently, and even we have identified different MSL1 MTPs.

Figure: minor error (shadow is a concern in image quality in presentation- it is appreciated if the author has an image without shadow background; also, the figure should be equally aligned, figure G is not aligned.

→Although we understand the reviewer's comment, we do not have other images at hand, and feel that these present the data clearly enough for the points made. The panels have been aligned and a new Figure 2 submitted.

L-233: 16-hour-light (remove the "-")
→fixed

L-243: ROS is a time sensitive assay, how the Author controls the ROS measuring among

different backgrounds in the msl2 msl3???? Such as staining, image acquisition, does the author did ROS measurement among different replication???

→As stated in the methods, the superoxide detection was performed for one hour, while hydrogen peroxide staining was performed overnight. After these time points, the staining solution was removed, and the first solution in the ethanol series added, effectively stopping the reactions. All tissues were incubated side-by-side so differences in incubation time between different backgrounds are negligible. We have revised the methods to clearly indicate this: "For superoxide detection, 21-day old seedlings of the indicated genotypes were collected and treated side-by-side. First, they were vacuum-infiltrated for 4 min . . ."

L- 270, L-273: roots/cm should be consistent format roots cm-1
→fixed

L-295-296: However, MSL1 did not interact with MSL2 or with MSL3, and MSL3 did not interact with itself (The Author should rewrite the sentence simple format rather than compound format as it's made abstruse for the reader)

→changed to "However, MSL1 did not interact with MSL2 or with MSL3. Also, MSL3 did not interact with itself."

L-304: (MSL1 (Lee et al., 2016)); (MSL2 and MSL3 (Haswell and Meyerowitz, 2006)) - similar error as L 205 to L-209
→fixed

L-317: (Carrie:2013kh; Xu et al., 2013) - Please double check the reference
→fixed

L-368 to L-373: (as defined in (Haswell, 2007)); (as defined in (Haswell and Meyerowitz, 2006)) (as defined in (Lee et al., 2016)); Similar error present as mentioned before (L-205 to L-209)
→fixed
